# On the existence of and mechanism for microwave-specific reaction rate enhancement†
†Electronic supplementary information (ESI) available. See DOI: 10.1039/c4sc03372h


**DOI:** 10.1039/c4sc03372h

**Published:** 2015-01-16

**Authors:** Gregory B. Dudley, Ranko Richert, A. E. Stiegman

**Affiliations:** a Department of Chemistry and Biochemistry , Florida State University , Tallahassee , FL , USA . Email: Stiegman@chem.fsu.edu; b Department of Chemistry and Biochemistry , Arizona State University , Tempe , AZ , USA

## Abstract

The use of microwave radiation to drive chemical reactions has become ubiquitous in almost all fields of chemistry. In all of these areas it is principally due to rapid and convenient heating resulting in significantly higher rates of reaction, with other advantages including enhanced product selectivity and control of materials properties. Although microwave heating continues to grow as an enabling technology, fundamental research into the nature of microwave heating has not grown at the same rate. In the case of chemical reactions run in homogeneous solution, particularly synthetic organic reactions, there is considerable controversy over the origins of rate enhancement, with a fundamental question being whether there exist microwave-specific effects, distinct from what can be attained under conventional convective heating, that can accelerate a reaction rate. In this Perspective, we discuss unique aspects of microwave heating of molecules in solution and discuss the origin and nature of microwave-specific effects arising from the process of “selective heating” of reactants in solution. Integral to this discussion is work from the field of dielectric relaxation spectroscopy, which provides a model for selective heating by Debye relaxation processes. The Perspective also includes a critical discussion of hypotheses of non-thermal effects (alternatively classified here as resonant processes) and an outline of specific reaction parameters for chemical systems in which microwave-specific Debye relaxation processes can result in observable reaction rate enhancement.

## Abstract 2

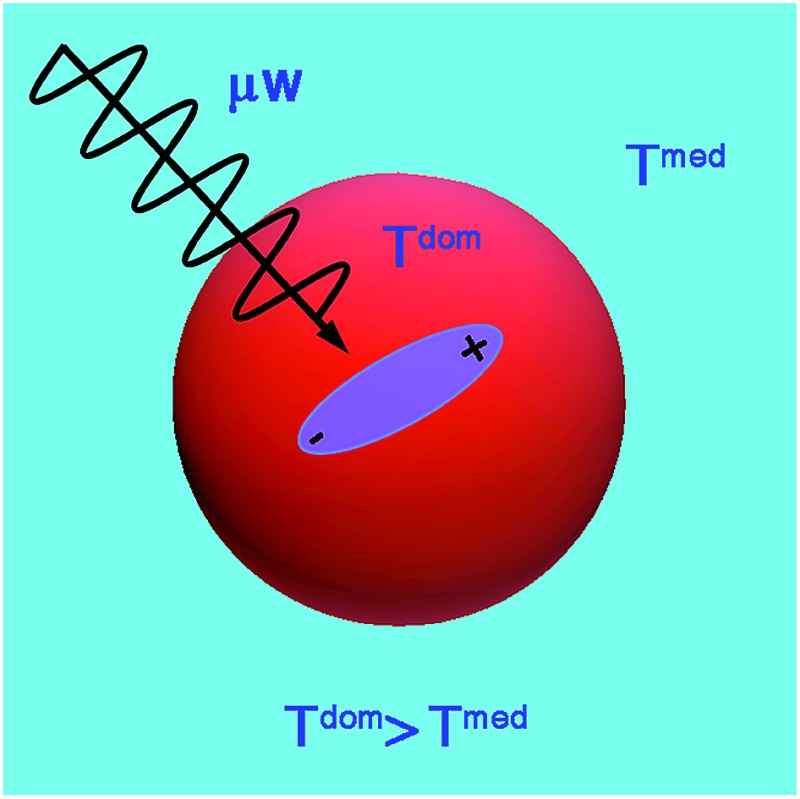
Microwave-specific chemical rate enhancement originates from the selective heating and accumulation of energy by solvated dipolar molecules in solution.

## 1. Introduction

An important and fundamental question in the field of microwave chemistry is whether an effect specific to microwave radiation exists that can lead to chemical reaction rates exceeding those realized through conventional heating to the same average temperature. While this hypothesis has been the source of continuing controversy, it has considerable and enduring appeal, as it suggests that it may be possible to obtain reaction products with a dramatic reduction of time and energy. The general observation that organic reactions run in microwave ovens can exhibit unexpectedly short reaction times, irrespective of any specific mechanism, was first reported in two pioneering studies carried out in the mid-1980s by Gedye and coworkers and Giguere and Majetich.^1,2^ The enhanced reactivity they observed proved general enough and potentially important enough to be termed “microwave-assisted organic synthesis” (MAOS) or microwave-organic reaction enhancement (MORE) chemistry.^3,4^ Since that time, the use of microwaves as an energy source in chemistry, and in organic chemistry in particular, has blossomed, and many aspects of it have been thoroughly reviewed.^5–10^

The attribution of this (apparently) enhanced reactivity to properties unique to microwave radiation emerged in several early studies in which reaction rates were examined under both conventional convective heating (*e.g.*, using an oil bath or heating mantle) and microwave heating made under (supposedly) comparable temperature conditions. In these studies, it was often found that microwave reactions proceeded at significantly higher rates than did the conventional reactions. In one of the earliest of these studies, Sun *et al.* observed a rate of hydrolysis of adenosine triphosphate molecules that was approximately 25 times faster under microwave radiation than under conventional heating at the same temperature.^11^ Subsequently, Bose *et al.* reported that the rapid synthesis of organic heterocycles in the microwave was due to microwave-specific processes other than simple thermolysis, and in studies of the Diels–Alder reaction between anthracene and diethyl maleate, Berlan *et al.* reported that the enhanced reaction rates observed in the microwave were examples of a specific microwave effect.^12,13^ Other studies followed in which it was hypothesized that the origin of observed rate enhancements was microwave-specific and due to unique interactions of microwave radiation with molecules in the reacting system.^14–17^ Ultimately, the physical processes associated with microwave-specific rate enhancement were segregated into two different phenomena: thermal effects, in which rate enhancements are caused by the generation of heat in the reaction medium due to the radiation field, and “non-thermal” effects, in which physical interactions between the reactant molecules and the electromagnetic field of the microwave radiation facilitate the bond-breaking and -making processes associated with a chemical reaction.

The vast majority of chemical reactions that undergo rate acceleration in the microwave are due to thermal effects arising from the extremely fast rate at which heat can be introduced in the bulk medium by the microwave. Rapid heating to high temperatures has even been shown to affect product selectivity in competitive reactions.^18^ Other related thermal processes that have been postulated for homogeneous systems are solvent superheating, nucleation-limited boiling, hot-spot formation, selective heating of specific reactants in solution, and elimination of so-called wall effects found in convective heating.^5,19,20^ When these effects are considered, it is generally believed that for reactions accelerated by the microwave, the increase in reaction rates is due to the temperature, and rates are not in excess of what would be expected for a reaction if the same temperature profile were attained conventionally. In many cases, however, these thermal mechanisms of rate enhancement can be thought of as “microwave-specific” in the sense that it is often difficult or impossible to produce them convectively.^21^ In the case of specific microwave effects, where rates in excess of those attainable through convective heating at a specific temperature are claimed, a non-thermal mechanism was often proposed.^22^ This has lead, in recent years, to the assumption that any and all claims of microwave-specific rate enhancement are, ipso facto, claims of a non-thermal effect.^23^

Not long after specific microwave effects were hypothesized, many of the studies on which that hypothesis was based were shown to be incorrect, in some cases by those who had initially proposed the effect.^24–27^ The basis of the rejection of virtually all of these claims was the fact that the temperatures in the microwave cavity were significantly underdetermined. When more accurate values were obtained, it was found that the reaction rates were indistinguishable from those of the conventional reactions run at that same temperature. This problem arose due to the difficulty in determining the correct temperature in the microwave cavity, a problem that was exacerbated by the use, in commercial research-grade microwaves, of an IR sensor that reported the temperature of the glass or quartz reaction vessel and not the solution itself. As it became clear that microwave-specific rate enhancements had been significantly over-reported, the validity of all such reports came into question. Notwithstanding this, reports of reaction rates in the microwave that are incommensurate with conventional experiments at a given temperature continue to be reported and explained by microwave-specific effects. A great many of these can be dismissed due to uncertainties in the measured temperature. In some few cases, however, summary rejection of the observations and/or conclusions is premature, either because the temperature measurements were done appropriately or the observations cannot be rationalized simply by assuming issues related to temperature measurement.^15,28,29^ The question raised by such studies is whether a physical mechanism does exist unique to microwave-driven reactions that can result in reaction rates exceeding those realized through convective heating at a specific temperature.

## 2. An alternative way of classifying microwave phenomena

The segregation of microwave effects into “thermal” and “non-thermal” processes is only somewhat meaningful in evaluating hypotheses of microwave effects, which had led to some confusion and debate over these classifications.^23^ In our opinion, a more significant distinction is between resonant and relaxation processes, of which only the latter is likely for solutions at typical microwave frequencies. If a dipolar molecule is unhindered and completely free to rotate in phase with the oscillating radiation field (*e.g.*, in the gas phase), then it will be in resonance when the frequency of the radiation coincides with the rotational frequency of the molecule. Quantum mechanically, this is when absorption of a photon occurs and the molecule is excited to a higher rotational state, as in microwave rotational spectroscopy. On the other hand, if the molecule is in a condensed medium with frictional damping, then its rotational motion will be out of phase with the radiation, and resonant processes give way to relaxation processes, as explained by Debye theory.^30,31^ For organic reactions in solution, any proposed mechanism of microwave-specific rate enhancement must be consistent with the physics of the relaxation process. Conversely, microwave-specific rate enhancements resulting from the direct absorption of radiation by resonant processes are unlikely.

Implicit in the “non-thermal” or resonant hypotheses is that microwave radiation can be absorbed by the system in such a way that it is not immediately converted into heat but instead induces a chemical reaction. Such processes are well known in conventional photochemistry where electronic excited states, which form from the absorption of UV and visible radiation, react at efficiencies that compete with those of radiative and non-radiative decay processes.^32^ For such a process to occur, however, there has to be absorption of the radiation by the molecules, which means that the energy required to create the excited state has to be available from the incident radiation. Commercial microwave ovens operate at a fixed frequency of 2.45 GHz (0.082 cm^–1^), which is significantly below (by many orders of magnitude) the energy required for exciting electronic or vibrational transitions. As such, there is no mechanism by which radiation can be absorbed to create an excited state that might lead to the activation of a chemical bond.

As noted above, molecules can absorb radiation in the microwave region of the spectrum in the gas phase to populate quantum mechanically defined rotational excited states. For small molecules, the lowest energy transitions typically occur at energies between ∼5–150 cm^–1^, with the frequency decreasing as the molecule gets larger.^33^ In going from isolated molecules in the gas phase to a completely condensed phase, the discrete rotational transitions broaden and drop to lower energy through intermolecular interactions. Water, for example, has discrete rotational bands in the gas phase appearing in an energy range from ∼19 to 122 cm^–1^, with its lowest energy transition occurring specifically at 18.57 cm^–1^ (556.5 GHz).^34^ In the condensed phase, the dielectric loss spectrum is broad and much lower in energy at 6.7–0.007 cm^–1^ (∼0.2–200 GHz) with a maximum at ∼18 GHz (0.6 cm^–1^).^35^

The restricted rotation of liquid-phase dipolar molecules in an oscillating field is a relaxation, not a resonant, process. It is typically treated by Debye theory, which is developed classically and for which the result of radiation absorption is the generation of heat. In fact, many of the arguments put forth in support of non-thermal or resonant effects invoke the classical Debye model—coupling of the field either to a net molecular dipole moment or to specific polar groups on the molecule—to “activate” the molecule toward a specific reaction. In most cases this is specious, as these proposed processes would actually be resonant processes that excite the molecule to a higher energy level though the absorption of a photon. As evidenced by the lack of resonance peaks in the dielectric spectra for frequencies below the THz range, there are no known molecular resonance processes that occur at the typical microwave oven frequency of 2.45 GHz, and any proposed mechanisms for non-thermal microwave effects that involve or require such a process are unlikely. For an independent molecule in the condensed phase, the current understanding is that microwave absorption can only result in a relaxation process that generates heat.

Thermal energy (heat) generated by such relaxation processes will ultimately be dispersed into the translational and vibrational degrees of freedom, but there is no mechanism to excite these degrees of freedom directly by a microwave radiation field. One of the hypotheses for non-thermal microwave effects is radiation-induced bond rotation (*i.e.*, functional group reorientation) and vibrational activation of attached polar functional groups (*e.g.*, hydroxyl, carbonyl) on a molecule that cause the group to be more reactive. As described above, such proposed rotational and vibrational activation will occur through thermal population of the normal modes in question, but they will not occur through direct absorption of microwave radiation, as such motion is part of a vibrational normal mode that is coupled to light in the infrared region.

Other hypotheses sometimes described as non-thermal microwave effects relate to differences in Arrhenius activation energies and pre-exponential terms between conventional and microwave-driven reactions. It has been suggested that the activation energy can decrease due to coupling of the dipole moment of a transition state along a reaction coordinate with the oscillating electric field.^36^ Microwave absorption processes arise from the coupling of radiation to permanent molecular dipole moments, and there is no reason why microwave energy would not couple to a transition-state or intermediate species with a permanent dipole. Such coupling would nominally result in a dielectric loss relaxation process to produce thermal energy associated with the transition-state or intermediate species in the solvent cage, potentially accelerating its passage over the activation barrier. In considering whether this represents a significant microwave enhancement pathway, the time frame of the process needs to be considered. For microwave radiation, there will be one complete peak-to-peak oscillation of the electromagnetic field every 0.4 nanoseconds. Transition state lifetimes are typically much shorter, usually in the sub-picosecond regime, so it is unlikely that the radiation field will generate enough localized heat in that period of time to contribute significantly to product formation.^37^ On the other hand, microwave-specific acceleration of a reaction because of relaxation processes linked to a longer-lived intermediate in the reaction path may be possible. We note that in the case of heterogeneous systems, where solid catalysts are absorbing the microwave radiation, there are well-demonstrated microwave effects that may be classified as “non-thermal”, though, importantly, the mechanisms are all consistent with specific dielectric loss processes. Of particular note are studies of microwave effects on the photocatalysis of TiO_2_ carried out by Horikoshi.^38,39^

For the reasons outlined above, postulates of non-thermal processes to explain the enhancement of reaction rates by microwaves are unlikely to be valid. These and other fundamental problems with the non-thermal effect hypothesis have been discussed thoroughly and lucidly in papers by Stuerga and Strauss, and the reader is directed there for a more complete dissertation.^36,40–42^

The fundamental physics of microwave interaction with molecules in solution, given in its simplest form by Debye theory, involves coercion of the dipole moment in condensed media. These interactions generate heat by non-resonant processes (*i.e.*, loss processes). Any microwave-specific acceleration of reaction rates must be related to the fundamental, heat-generating, dielectric loss process. Unfortunately, the many erroneous claims of microwave-specific rate enhancements in organic reactions have led some to conclude that such rate accelerations are either impossible or impractical because “heat is heat” regardless of how it is generated.^43^ While this position is understandable, it belies the fact that microwaves heat through a profoundly different mechanism than occurs in conventional convective heating. It is certainly true that many of the advantages of microwave heating in synthetic chemistry relate to rapid, efficient, and volumetric bulk heating. If the temperatures and heating rates could be properly matched, then there would be no difference in the reaction rates in most cases. However, it is also true that selective heating of a molecule possessing a high absorption cross section is possible (*cf.* “molecular radiators”^44^). Such selective heating is consistent with the fundamental physics of microwave heating and can in principle lead to reaction rates exceeding what would be observed in conventional experiments conducted at the same bulk temperature (*i.e.*, the measured temperature of the medium).^9,45,46^

In particular, if a highly absorbing molecule is present as a solute in a non-absorbing solvent, then the heat gained by the entire solution must arise from convective heating of the solvent medium by the absorbing solute as it absorbs radiation. Under steady-state conditions in which radiative heating of the absorber is followed by convective heat flow into the medium and, ultimately, out of the system, the effective temperature (*i.e.*, energy) of the absorbing species will necessarily be, on average, higher than that of the solvent. If the absorbing species is a reactant in a chemical transformation, then based on the temperature-dependences of the Arrhenius activation energy and pre-exponential term, faster reaction rates should be realized. Such rate acceleration will depend on the amount of heat stored in the absorbing molecule, which will, in turn, depend on various parameters of the system. The paradigm of selective heating is both common and uncontroversial in heterogeneous systems such as a microwave-absorbing heterogeneous catalyst where the catalyst can be selectively heated to temperatures much higher than the surrounding medium.^47,48^

## 3. Theoretical considerations of microwave heating

In the macroscopic limit, the conversion of absorbed microwaves into heat in a dielectric (*i.e.*, nonconductive) material is described through the dielectric continuum model.^9,49–51^ In this treatment, the frequency dependent bulk permittivity of a material, *ε*, is expressed in its complex form

1 *ε̂*(*ω*) = *ε*′(*ω*) – *iε*′′(*ω*),

where *ε*′ is the storage component (which approaches the static dielectric constant in the limit of low frequencies) and *ε*′′ is the dielectric loss. Both the real and imaginary components of the permittivity are frequency-dependent and connected by the Kramers–Kronig relation, with *ε*′ and *ε*′′ gauging the amount of energy that can be stored or irreversibly converted to heat in the material, respectively.

For molecules in solution, the permittivity reflects polarizations *P* that occur *via* the interaction of charges with the oscillating electric field, *e.g.*, at microwave frequencies. The total polarization can be broken down into several polarization contributions that differ in amplitude and frequency dependence, as shown in eqn (2).

2 *P* = *P*_dipolar_ + *P*_ionic_ + *P*_electronic_.

The constitutive equation relating polarization *P* and field *E* define the susceptibility *χ* and the permittivity *ε*, which for the steady state case reads

3 *P* = *χε*_0_*E* = (*ε* – 1)*ε*_0_*E*,

with *ε*_0_ being the permittivity of vacuum. The electronic polarization contribution, *P*_electronic_ in eqn (2), represents the distortion of the electron cloud of the constituent atoms or molecules by the electric field. This distortion creates an induced dipole in the materials; however, this process does not contribute to heating since the polarization is rapid and thus remains in phase with the applied field (*i.e.*, no loss process exists). Notably, this type of polarization is often invoked to support various non-thermal, microwave-specific effects; however, such interactions do not transfer energy to the material and therefore will not contribute to bond-making and -breaking processes. In the case of ionic solutions, the electric field will induce a net polarization, *P*_ionic_, in the medium through displacement of the positive and negative ions relative to each other in the solution. Due to the viscous drag of the solvent and solvation shell, ion movement will not respond in phase with the radiation, resulting in a loss component (*ε*′′ > 0) and eventually in heating. The large magnitude of this polarization mechanism is why salt solutions heat so efficiently. For molecules possessing a permanent dipole moment, the polarization induced by the oscillating field, *P*_dipolar_, which occurs through the torque on the permanent dipole moment exerted by the oscillating electric field, will tend to orient the molecule in the direction of the applied field. The energy loss and concomitant heating are caused by frictional losses that occur during the reorientation or rotational diffusion of the dipoles. In short, it is the dipolar relaxation process that converts the work done by the field to heat. All of these polarization processes can be expressed in terms of their real and imaginary components corresponding to the in-phase (storage) and out-of-phase (loss) process, respectively. Regardless of the nature of the loss process, the average power density *p* absorbed from a field *E*(*t*) = *E*_0_ sin(*ωt*) is given by4
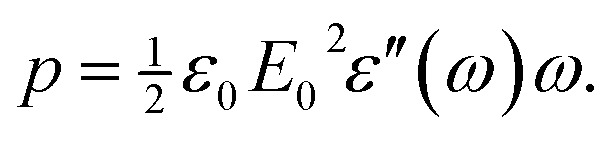



In the hydrodynamic limit, the frequency dependence of the polarization originating from dipole reorientation follows Debye's dielectric relaxation theory, which yields eqn (5):5
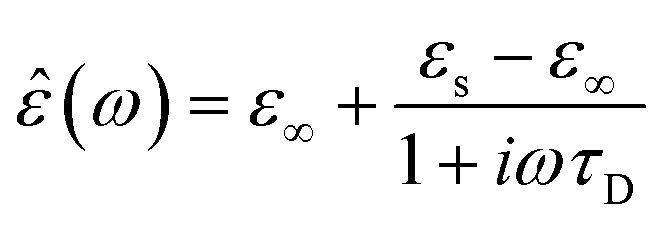
where *ε*_∞_ is the permittivity at the limit of infinite frequency, *ε*_s_ is the permittivity under a static electric field, and *ω* is the frequency of the applied radiation.^31^ The time constant of the relaxation processes, *τ*_D_, characterizes the time required for the field-perturbed system to return to equilibrium. In the case of dipolar reorientation, this will be the time it takes the dipoles to lose memory of the orientation it had at time zero. The frequency dependence of the real and imaginary parts of the permittivity of eqn (5) is shown in eqn (6) and (7), respectively.6
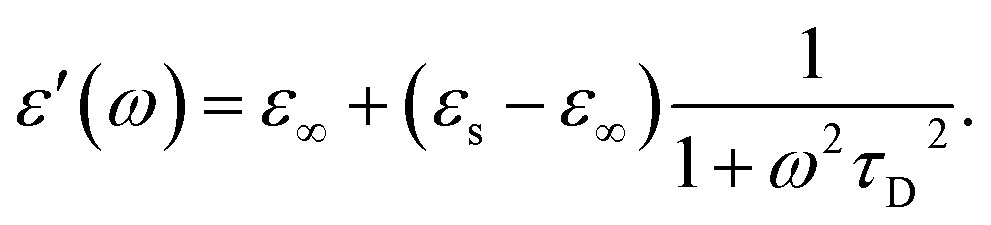

7
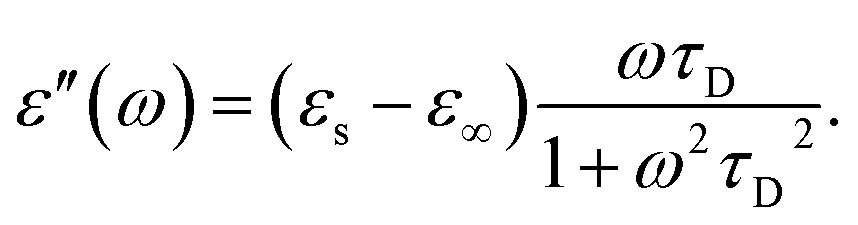



Energy dissipation in a dielectric medium is often quantified by the loss tangent (tan *δ*) or dissipation factor (D), which can be obtained from permittivity *via* the ratio of loss over storage component according to

8 tan *δ* = *ε*′′/*ε*′.

## 4. The mechanism of selective heating

**Fig. 1:**
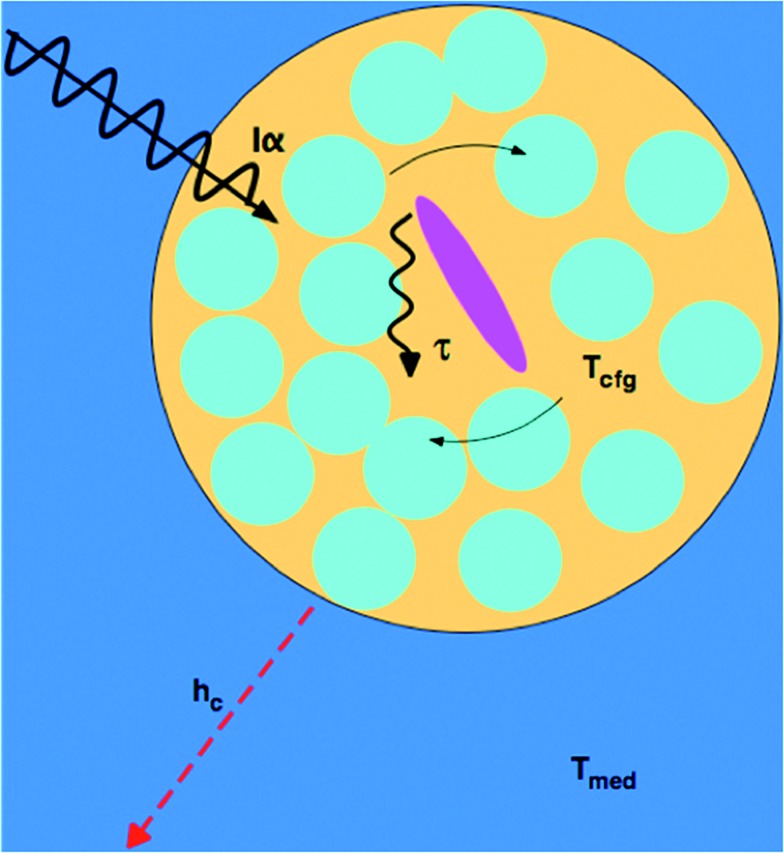
Microwave-absorbing domains composed of an absorbing dipolar molecule (magenta) in a non-absorbing solvent cage (blue). The microwave intensity, *I*, is absorbed with a cross section *α*, which excites a configurational change (curved solid arrows) that relaxes (wiggly arrow) with a time constant *τ*_T_ yielding a transient configurational temperature, *T*_cfg_ > *T*_med_, if the heat dissipation through convective heat transfer, *h*_c_, to the medium at a temperature, *T*_med_, is slow.

A more microscopic understanding of microwave heating in terms of dielectric relaxation processes in liquids has been provided in studies by Richert and coworkers.^45,46,52^ For pure liquids close to their glass transition temperature, dynamically distinct domains are created, the dimensions of which have been estimated at 1–3 nm. The dielectric behavior of each domain follows eqn (5), but site-specific values of *τ*_D_ vary by orders of magnitude. As a result, the absorption of electromagnetic radiation of a given frequency *ω* varies spatially, rather than being homogeneous, because efficient absorption requires *ωτ*_D_ ≈ 1. The absorption process excites configurational modes within the domains; once excited, these modes undergo a configurational relaxation process characterized by a time constant, *τ*_T_, not far from the structural relaxation time of the liquid. After configurational relaxation has transpired, the energy is dissipated as heat into the phonon bath of the surrounding medium. In viscous liquids, this feature localizes the energy for times that are orders of magnitude longer than what is expected on the basis of the thermal conductivity. During the time of this excess configurational temperature, molecules are more mobile, as if the local temperature were higher than that of the phonon bath. By analogy, highly absorbing solutes create a similar heterogeneity regarding absorptivity, and various factors limit the rate at which the locally absorbed energy contributes to the average sample temperature: the configurational relaxation time *τ*_T_, barriers to thermal conduction from solute or solvation shell to the bulk solvent, and the finite thermal conductivity of the bulk solvent itself. At microwave frequencies, the generation of local net temperatures greater than that of the macroscopic average constitutes a radiation specific heating process, regardless of whether excess local heat or configurational excitation is at the origin of accelerating the reaction. In the case of highly absorbing dipolar molecules dissolved in nonpolar aromatic solvents, the domains are well defined chemically by the dipolar molecule and the solvation sphere around it, as illustrated schematically in Fig. 1.^53^ Such discrete domain formation is related to the heterogeneous domains formed in pure liquids, as discussed above, because a small subset of the sample will absorb a majority of the power in a spatially heterogeneous fashion. As illustrated in Fig. 1, the microwave radiation couples with the permanent dipole moment, adding energy to that subset as work, which is subsequently transformed to heat.

The amount of energy absorbed by a domain will depend on the microwave flux and the absorption cross section of the domain, *Iα*, at the applied frequency (2.45 GHz). Within the domain, dielectric loss occurs mainly through the reorientation of the solute dipoles within the solvent shell. These relaxation processes occur with time constants *τ*_T_, which, for this type of configuration relaxation, is inherently slow. The fact that the frequencies of microwave radiation excite configurational modes that relax slowly distinguishes it from conventional heating in that the direct transfer of energy to the rapid (predominantly vibrational) phonon modes of the medium is inherently inefficient (*i.e.*, small heat transfer coefficient, *h*_c_).^45,46^ As such, heat will accumulate in the domain containing a solute, giving the molecules contained within it a temperature (*T*_cfg_) that can be larger than that of the surrounding medium (*T*_med_). The higher the configurational temperature relative to the measured temperature of the bulk medium, the more pronounced the specific microwave effect should be, providing one or all of the absorbing species is a reactant.

The primary question has been; are there conditions under which sufficient heat will be stored in the domains to generate measurable rate enhancement? A number of factors will potentially affect this, including the magnitude of microwave absorption, which is controlled by both molecular and solvent parameters, and the efficiency of convective heat transfer out of the domain, which is affected by the thermal conductivities and heat capacities of the solute – solvation shell – solvent system. Moreover, in a real microwave system, convective heat transfer out of the container will also play a role. Heat transfer coefficients are typically a function of Δ*T*, which for dipolar molecules in a solvent shell is the difference in effective temperature between the domain (*i.e.*, configurational temperature) and the medium. During the initial stages of heating, energy will be stored, as heat transfer out into the medium will be slow. As heating continues, however, the heat transfer rate out of the domain will ultimately decrease as the medium heats up and Δ*T* gets smaller. As such, the amount of heat stored will vary dynamically over the course of the microwave irradiation time.

For selective microwave heating effects to be observed in chemical processes, it is necessary to find experimental conditions that optimize heat storage in the domains. For example, based on the arguments given above, it is clear that systems where the solvent itself is a strong microwave absorber along with the reactants will be unlikely to yield significant heat storage; if both the solvent and reactant are heated simultaneously, then Δ*T* will be small over the course of the heating process. For that reason, reports of reactions in polar solutions (*e.g.*, DMSO) that seem to show a specific microwave effect may be problematic. The reaction conditions most likely susceptible to selective microwave heating are those in which the solvent is non-absorbing while the reactant(s) are strongly absorbing. Beyond this, there are other properties of molecular absorbers and their medium that can be adjusted to enhance microwave absorption properties and, ultimately, energy storage.

A general approach that we have followed in designing systems where heat accumulation in the domains might be maximized, and microwave-specific rate enhancement observed, is to optimize the system (reactant, solvent, vessel, *etc.*) for maximum differential microwave absorption using the molecular and solvent parameters implicit in the Debye treatment of dipolar relaxation. Large microwave absorption by the reactant(s) is a necessary, though not necessarily sufficient, condition for heat accumulation in the domains.

## 5. Molecular and solvent parameters affecting selective heating

**Fig. 2:**
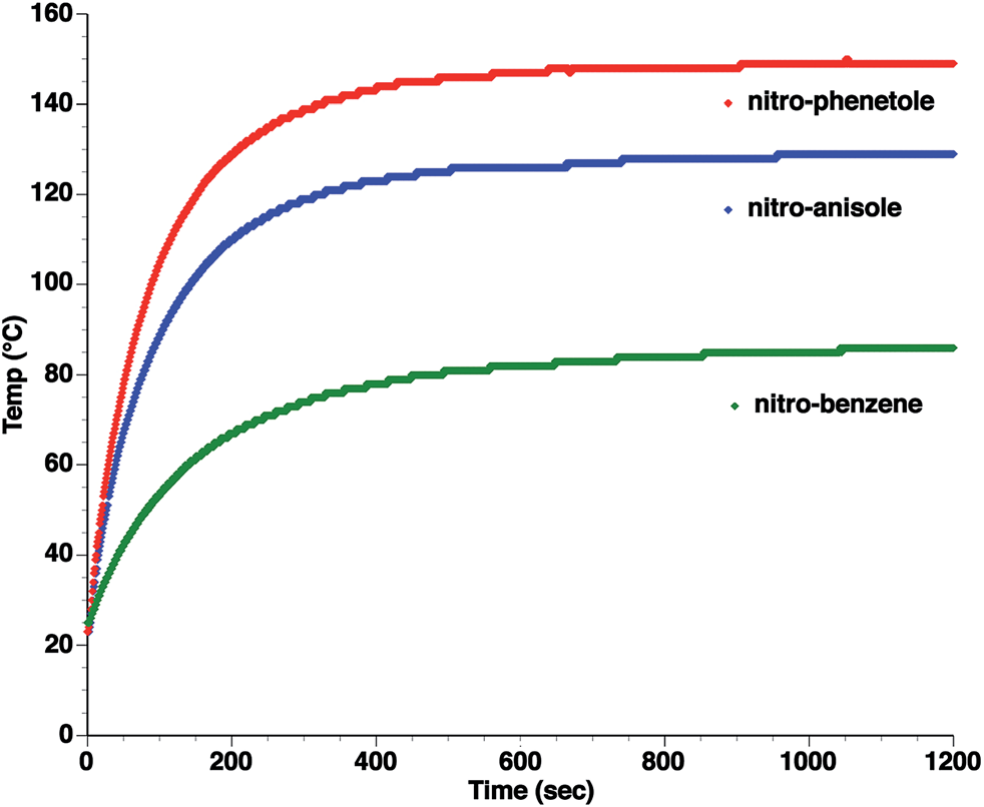
Microwave heating curves at 50 W irradiation for a series of dipolar molecules (0.1 M) in non-absorbing solvent (mesitylene). Note that the heating curves were collected under open vessel conditions.

For the case of a dipolar compound in a non-absorbing solvent, the Debye and Guggenheim equations provide a simple relationship (eqn (9)) between the incremental dielectric loss (*ε*′′inc) due to the absorbing species and its electric dipole moment, *μ*, the relaxation time, *τ*_D_, and the concentration, *n*, in moles per unit volume^54,55^
9
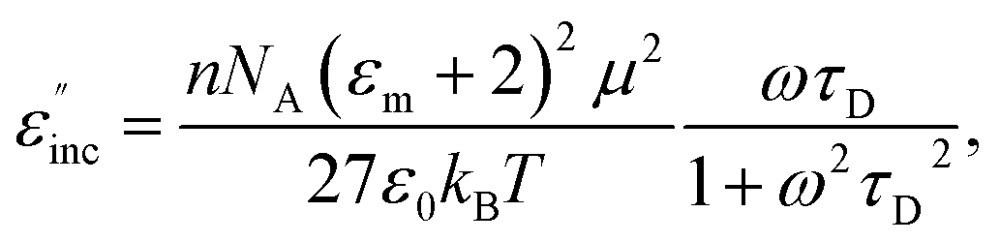
where *ε*_m_ is the permittivity of the medium (solvent), *N*_A_ is Avogadro's constant, and *ε*_0_ is the permittivity of vacuum. While the use of eqn (9) for the quantitative determination of dielectric loss is not reliable, the intrinsic structure–property relationships contained in the equation can often be observed in the case of microwave heating. Heating curves at 50 W applied microwave power for a series of structurally similar aromatic dipolar molecules (0.1 M) in a non-absorbing solvent (mesitylene) are shown in Fig. 2.

As can be seen, the heating rate increases with the dipole moment (Table 1), indicative of an increase in the dielectric loss. The observed heating curves follow the usual trajectory of such measurements where an early linear increase in temperature is observed, followed by a leveling off when the rate of heat flow out of the system equals the rate of heat formation in the system, and steady-state conditions are established. Using the early linear stages of the heating curve (20–60 s), before the heat flow out of the system becomes significant, a heating rate for each type of solute can be determined (Table 1). Using the heating rate, mass, and heat capacity of mesitylene at room temperature (213 J mol^–1^ K^–1^), the power emanating from this series of “molecular radiators” into the non-absorbing solvent during irradiation can be mathematically estimated (Table 1). The heating power of the molecular radiators was estimated to range from 3.83 kW mole^–1^ for nitrobenzene to 9.97 kW mole^–1^ for *p*-nitrophenetole (ESI†).

**Table 1:** Power produced by molecular radiators roughly correlates with dipole moment

	*μ* (D)	dT/dt (°C s^–1^)	kW mole^–1^
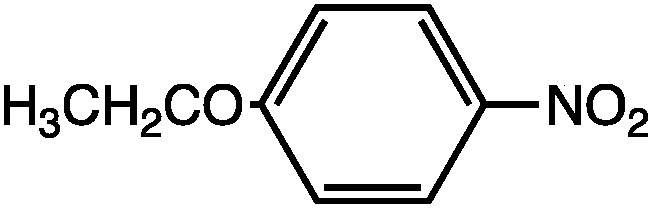	5.81	0.652	9.97
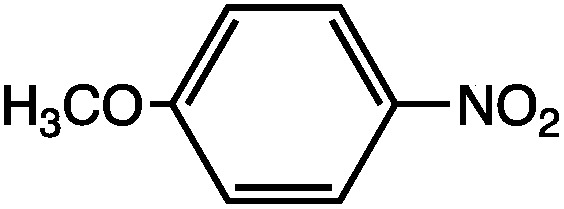	5.61	0.531	8.13
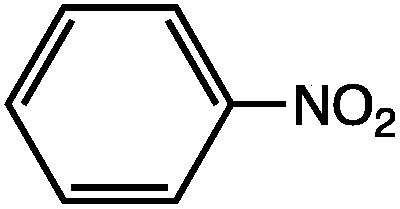	4.39	0.251	3.83

Although the power determinations do not indicate how much heat is actually stored in the domains around the dipole, it is clear that molecular properties such as the dipole moment have a profound effect on the magnitude of the heat being generated. Therefore, systems optimized in such a way are likely to realize a higher configurational temperature in the domains.

The other factor that affects the dielectric loss is the relaxation time, *τ*_D_. Eqn (7) predicts that the relaxation time *τ*_D_ impacts the absorption by changing the loss *ε*′ at the frequency *ω*, with the maximum occurring at *ωτ*_D_ = 1. The effect of molecular and solvent properties on the relaxation time was given by Debye (eqn (10)) in the rotational hydrodynamic approximation.10
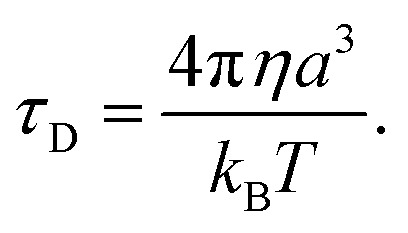



In this approximation, the lifetime will increase with the increasing viscosity (*η*) of the solvent and with the volume (*a* ^3^) of the absorbing molecule. The hydrodynamic approximation provides, at best, a qualitative prediction of the changes in relaxation time as a function of viscosity, but it often fails quantitatively due to the fact that, at a microscopic level, there can be many specific interactions between the dipolar molecule and the solvent that are not well described using a continuum description of the solvent. Likewise, the volume, *a* ^3^, will not necessarily correspond simply to the molecular dimension of the probe molecule, and will depend, in part, on the relative size between the dipolar molecule and the solvent.^53^

**Fig. 3:**
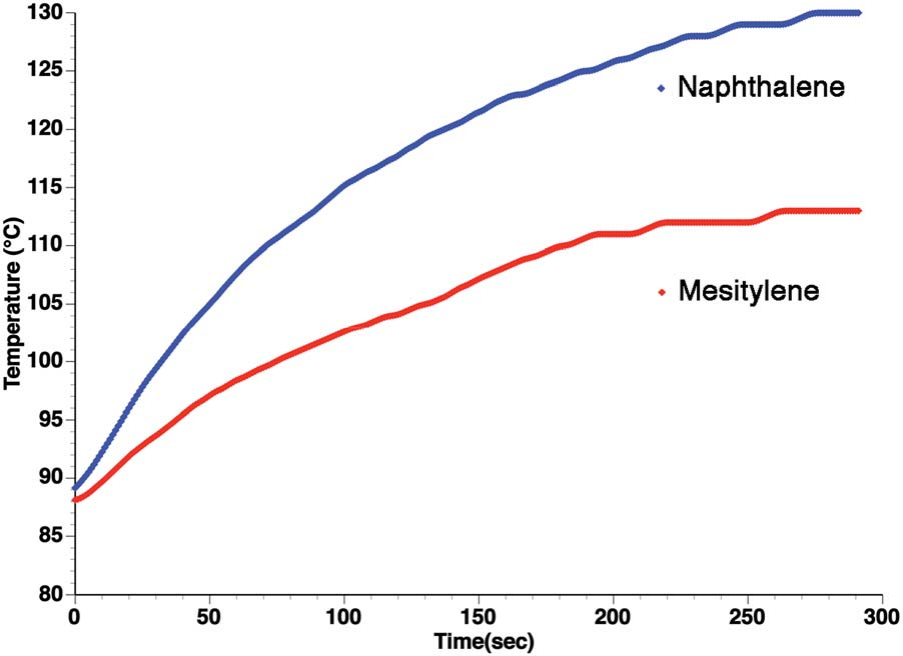
Heating curves at 25 W applied power for *p*-nitrophenetole (0.1 M) in (blue) naphthalene and (red) mesitylene. Note that heating was initiated above the melting point of naphthalene under open vessel conditions.

From the standpoint of optimizing microwave absorption properties, eqn (10) predicts that changing the viscosity of the nonpolar solvent should modify the microwave absorption *via* a change in *τ*_D_. This could explain the increase in heating rate that is often observed in going to a more viscous solvent. This can be seen in Fig. 3, which shows the heating curves collected at 25 W of applied power for *p*-nitrophenetole dissolved in liquid naphthalene and mesitylene, which have viscosities of ∼0.75 and ∼0.52 cP, respectively.

What is clear from these illustrations is that specific reactant and solvent parameters can be effectively “tuned” to maximize the conversion of microwave energy to heat. Obviously, this heating efficiency does not necessarily guarantee sufficient heat accumulation in the solute domains to accelerate chemical reactions in a meaningful way; that will depend on a number of additional thermodynamic parameters of the system. It is reasonable to suggest, however, that systems optimized for microwave absorption provide a logical place to start in investigating and, perhaps, exploiting the selective heating process. More studies are needed to guide this optimization process. Notably, there have been at least two attempts to measure the effective temperature of absorbing molecules under microwave irradiation using *in situ* Raman spectroscopy.^56,57^ One study focused on benzaldehyde in hexanes, and the other focused on neat DMSO. In neither case was any significant selective heating observed, although of course no selective heating would be expected in neat DMSO. Based on our observations, additional optimization of the focal system—including more polar solutes in a wider array of nonabsorbing solvents—may enhance the probability of selective heating in a way that might be measurable by *in situ* Raman spectroscopy.

Our approach has focused on the hypothesized impact of selective heating on chemical reaction kinetics. We designed and optimized two organic reaction systems according to the parameters outlined above. In both cases, measurable microwave specific rate enhancements were ultimately observed. These studies establish unambiguously that microwave-specific rate enhancements are possible, and they define some, but certainly not all, of the experimental designs and conditions that can enhance the effect. The results were largely interpretable in terms of the domain model for microwave heat accumulation.11
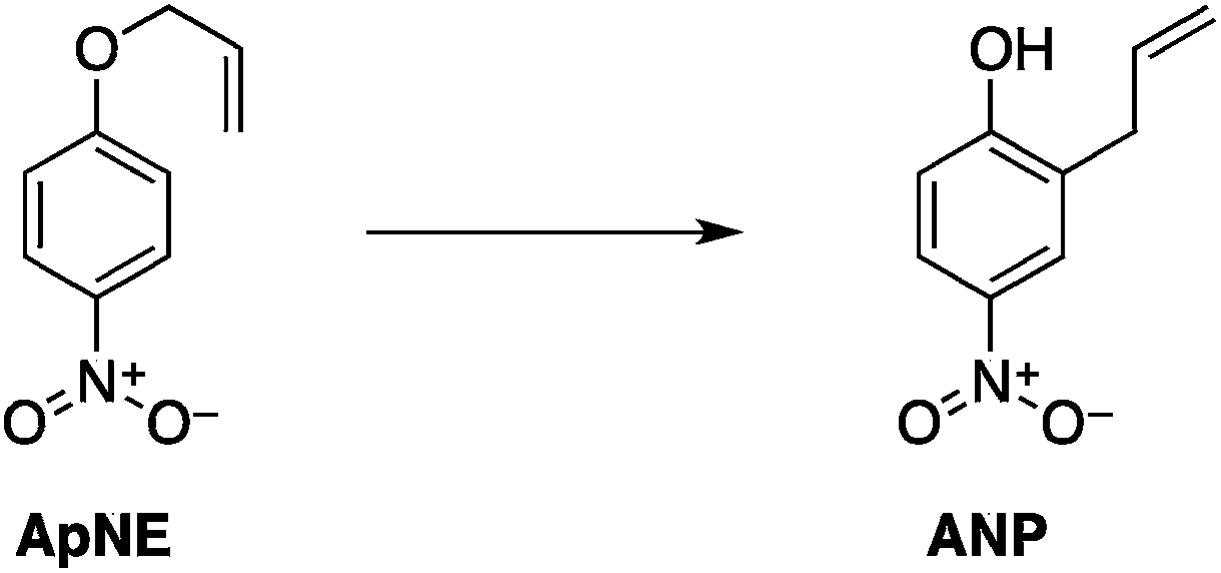

12
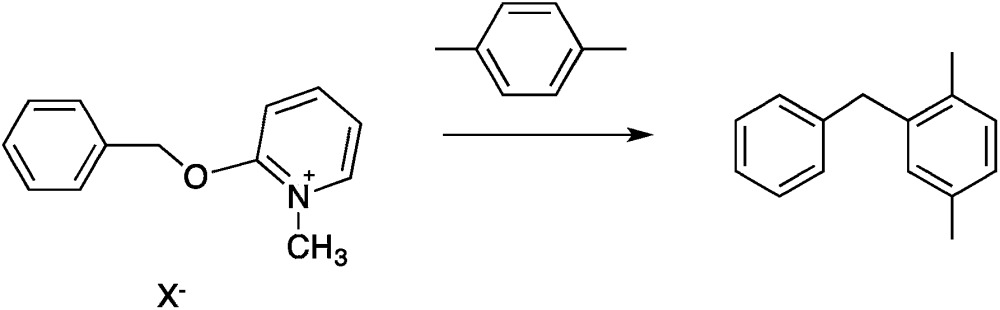



Two reactions were studied: the thermal aryl Claisen rearrangement of allyl *p*-nitrophenyl ether (rxn. (11)) and the thermal Friedel–Crafts benzylation of arene solvents (*e.g. p*-xylene) using an ionic benzyl-transfer reagent, 2-benzyloxy-1-methylpyridinium tetrakis[3,5-bis(trifluoromethyl)phenyl]borate (rxn. (12)).^44,58,59^ In both systems non-polar, non-absorbing solvents were used. For the aryl Claisen, addition of the nitro group in the para position in allyl phenyl ether increased the dipole moment and microwave absorption cross-section; allyl phenyl ether itself shows minimal microwave heating. Heating curves were measured in different solvents, and the one that gave the highest heating rate, naphthalene, was used in the reaction. For the Friedel–Crafts study, the ionic benzylating agent is strongly microwave absorbing; the lipophilic borate counterion was selected to enhance solubility in organic solvents. The solvent, *p*-xylene, was chosen largely because benzylation produces a single product at a convenient rate.

The experimental approach by which the existence and magnitude of microwave-specific effects was assessed relied on the measurement of reaction kinetics (conversion over time) in both conventional and microwave-heated experiments. Measurement at different temperatures under conventional heating provided a precise determination of Arrhenius parameters for the reactions. Kinetic data were collected under microwave heating with the bulk temperature of the reaction solution carefully measured *in situ* using a fiber optic thermometer. From the reaction rates under microwave heating, we could determine the effective temperature of the reactant, *i.e.*, the temperature that would produce the same reaction rate if the reaction were carried out conventionally. We reasoned that if the effective temperature was greater than the measured temperature of the bulk solution in the microwave, then there must be a microwave-specific rate enhancement. The magnitude of the enhancement could then be determined quantitatively from the ratio of the calculated rate constants for the microwave and conventional experiments at the measured temperature of the microwave solution.

The magnitude of the microwave-specific rate enhancement was generally less than one order of magnitude; more subtle than certain historical claims. It was also highly dependent on specific reaction conditions (reactant concentration, solvent, *etc.*) in ways that had not been described previously. More importantly, the magnitude of the rate-enhancement varied based on how the microwave radiation was applied, as follows. Under constant temperature conditions, which are conditions under which many microwave reactions are run, little or no rate enhancement was observed. Conversely, under conditions of constant applied power, rate enhancements of two- to four-fold or greater were observed. Finally, the most significant microwave-specific rate enhancement occurred under pulsed power conditions, where a sequence of applied microwave pulses, at high constant power, were used to drive the reaction through a series of rapid heating and then cooling cycles. Rate enhancements approaching an order of magnitude or more were observed under these conditions.

What is clear from the above discussion and the recent experimental results is that a mechanism exists, inherent in the fundamental physics of microwave heating, whereby molecules can be selectively heated in solution to produce microwave-specific rate enhancements. Experimental observations can be rationalized in terms of a selective heating model in which heat is generated and transiently stored in the domain defined by the solvated molecule and its surrounding solvent cage. This energy storage mechanism leads to enhancements in chemical reaction rates that are incommensurate with the temperature of the bulk medium. It is also clear from the experimental results that the chemical factors affecting the magnitude of the effect are complex, and considerably more research is needed to improve our understanding of the origins of these effects.

## Conclusions

Current chemical kinetics and molecular dynamics theory has developed over many decades based on the physics of conventional convective heating (*e.g.*, Arrhenius kinetics). In most cases, these theories can be adequately applied to explain chemical dynamics under microwave heating. However, under certain conditions, the unique heating properties of microwave radiation produce solution-phase dynamics that cannot be rationalized solely in terms of current physical organic theory based on the measureable temperature of the bulk solution. For chemical synthesis, these deviations from conventional heating provide opportunities to enhance the utility of microwave heating above current practices, perhaps leading to reduced energy costs of reactions and processes, refined and/or altered reaction pathways and equilibria, and maybe even identification of new modes of reactivity unique to microwave heating. Such opportunities have yet not been well established in multi-component (macroscopically homogeneous) solution, although they are well precedented in macroscopically heterogeneous systems. Chemists are adept at exploiting subtle energy changes to gain advantages in synthesis; better understanding of the subtle influences of microwave heating on chemical dynamics has the potential to make significant impacts on the molecular sciences.
