# Supplementary material for: On the existence of and mechanism for microwave-specific reaction rate enhancement
Source: Chem Sci. 2015 Jan 16;6(4):2144–52. doi: 10.1039/c4sc03372h (PMC5639434; doi:10.1039/c4sc03372h)
Supplement: Supplementary file 1 [file SC-006-C4SC03372H-s001.pdf]

This journal is © The Royal Society of Chemistry 2015

Supplementary Material

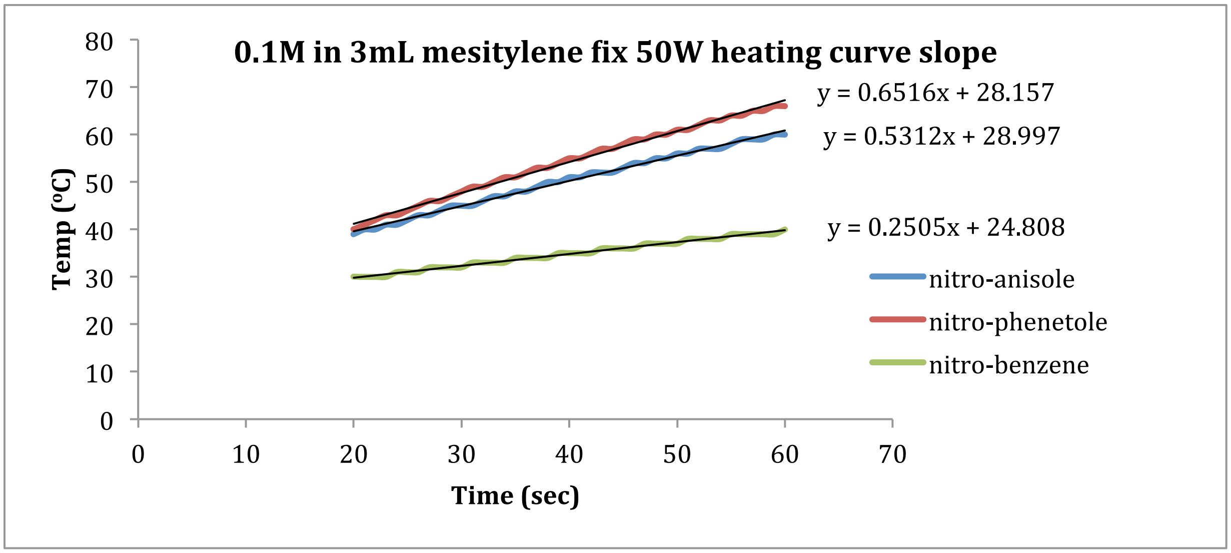

The

$$\text{moles of solute} = 3.00 \times 10^{-4}$$

$$\text{moles of mesitylene} = 2.165 \times 10^{-2}$$

$$\mathrm{C}_p = 213 \text{ J/mol K for mesitylene}$$

$$\mathrm{P(W/mole)} = \frac{\left(\frac{dT}{dt}\right)(\mathrm{C}_p)(n_{\text{moles of solvent}})}{n_{\text{moles of solute}}}$$
